# Caspase-8 inhibition represses initial human monocyte activation in septic shock model

**DOI:** 10.18632/oncotarget.9648

**Published:** 2016-05-26

**Authors:** Maria Jose Oliva-Martin, Luis Ignacio Sanchez-Abarca, Johanna Rodhe, Alejandro Carrillo-Jimenez, Pinelopi Vlachos, Antonio Jose Herrera, Albert Garcia-Quintanilla, Teresa Caballero-Velazquez, Jose Antonio Perez-Simon, Bertrand Joseph, Jose Luis Venero

**Affiliations:** ^1^ Department of Biochemistry and Molecular Biology, Faculty of Pharmacy, Universidad de Sevilla, Sevilla, Spain; ^2^ Department of Oncology-Pathology, Cancer Centrum Karolinska, Karolinska Institutet, Stockholm, Sweden; ^3^ Instituto de Biomedicina de Sevilla (IBiS)-/CSIC/ Universidad de Sevilla, Sevilla, Spain

**Keywords:** sepsis, monocyte, inflammation, caspase-8, necroptosis, Pathology Section

## Abstract

In septic patients, the onset of septic shock occurs due to the over-activation of monocytes. We tested the therapeutic potential of directly targeting innate immune cell activation to limit the cytokine storm and downstream phases. We initially investigated whether caspase-8 could be an appropriate target given it has recently been shown to be involved in microglial activation. We found that LPS caused a mild increase in caspase-8 activity and that the caspase-8 inhibitor IETD-fmk partially decreased monocyte activation. Furthermore, caspase-8 inhibition induced necroptotic cell death of activated monocytes. Despite inducing necroptosis, caspase-8 inhibition reduced LPS-induced expression and release of IL-1β and IL-10. Thus, blocking monocyte activation has positive effects on both the pro and anti-inflammatory phases of septic shock. We also found that in primary mouse monocytes, caspase-8 inhibition did not reduce LPS-induced activation or induce necroptosis. On the other hand, broad caspase inhibitors, which have already been shown to improve survival in mouse models of sepsis, achieved both. Thus, given that monocyte activation can be regulated in humans via the inhibition of a single caspase, we propose that the therapeutic use of caspase-8 inhibitors could represent a more selective alternative that blocks both phases of septic shock at the source.

## INTRODUCTION

Historically, sepsis represented one of the major causes of death until the discovery of antibiotics in the early 20th century. However, emerging diseases such as Ebola, H5N1 avian influenza or SARS, together with an increase in antibiotic resistance in previously controllable pathogens such as *Mycobacterium tuberculosis* or MRSA have led to sepsis once again becoming a global burden [1, 2]. This is due to the high mortality rates associated to severe sepsis, ranging from 20% to 80% in the event of a systemic inflammatory response syndrome (SIRS) [3]. Although SIRS has generally been associated with sepsis, it can also occur in non-infectious conditions such as trauma, burns or severe injuries. This is due to the fact that SIRS is initiated not only by the detection of pathogen-associated molecular patterns (PAMPs) but also damage-associated molecular patterns (DAMPs) [4, 5]. The recognition of these molecules by monocytes and neutrophils in the bloodstream leads to uncontrolled activation, proliferation and the release of cytokines [6–8]. The resultant cytokine cascade triggers an initial pro-inflammatory state that leads to generalized inflammation and multi-organ failure. This inflammatory state also causes lymphocyte and dendritic cell apoptosis, and deterioration of phagocyte function. The combined depletion and impaired activation of immune cells ultimately leads to an immunosuppressive state, which has been hypothesized to cause both the high mortality and the long-term side effects of sepsis [9, 10]. Furthermore, given the positive feedback nature of the resultant response, the irreversible cytokine storm can continue even after the clearance of any pathogen, complicating the treatment and thus worsening the prognosis [4, 11].

To date, there are no drugs specifically approved for the treatment of SIRS in humans. Clinical trials have mostly been based around decreasing the pro-inflammatory response by developing strategies to neutralize cytokines such as neutralizing antibodies or thrombogenic products. These strategies however cannot prevent the onset of the immunosuppressive state, and so current research is also focusing on apoptosis inhibitors to prevent lymphocyte and dendritic cell depletion [12–15]. Given that monocytes are the upstream cells in the process, as well as important modulators of the innate immune response, the regulation of monocyte activation could represent an alternative in the treatment and prevention of SIRS [16].

We therefore sought to further understand the mechanism of activation of blood monocytes. Previous studies have shown that multiple members of the caspase family of proteins are involved in regulating this process. Caspases, or cysteine-aspartic proteases, are most well known for their roles in apoptotic cell death [17]. However, it is becoming clear that they exhibit additional non-apoptotic roles, especially in cells of the myeloid lineage. For instance, caspase-8 has been shown to be critical for the differentiation of monocytes into macrophages [18–20]. Furthermore, it has been demonstrated that caspases 8, 3 and 7 play an important role in the neurotoxic pro-inflammatory activation of microglia [21, 22]. In line with this, caspase-8 inhibition led to decreased microglial activation and neuronal damage associated to inflammation. Finally, caspase-8 has been shown to inhibit a type of cell death termed ‘programmed necrosis’ or necroptosis by cleaving receptor-interacting protein kinase 1 (RIPK1) [23–25]. Necroptosis, or programmed necrosis, occurs downstream of RIPK1 activation, via oligomerization with receptor-interacting protein kinase 3 (RIPK3) and subsequent activation of mixed lineage kinase domain-like protein (MLKL), which drives membrane disruption [26]. Since necroptotic cell death leads to the release of DAMPs from cells, the role of caspase-8 in inhibiting this process may also be important in controlling the activation of innate immune cells [27].

Here, we aimed to understand the role of caspase-8 as a regulator of monocyte activation and its potential as a target for the control of SIRS. We found that caspase-8 regulates human monocyte activation and caspase-8 inhibition results in decreased monocyte activation and cytokine release. Furthermore, we also observed that caspase-8 inhibition of activating monocytes promotes their death by necroptosis, which could potentially reverse the accumulation of monocytes observed in SIRS. Similar results were achieved in mouse monocytes treated with broad caspase inhibitors, which have previously shown promise in murine models of septic shock. Together, these results suggest that caspase-8 inhibitors could be used in the prevention of SIRS.

## RESULTS

### Caspase-8 regulates human monocyte activation

**Figure 1:**
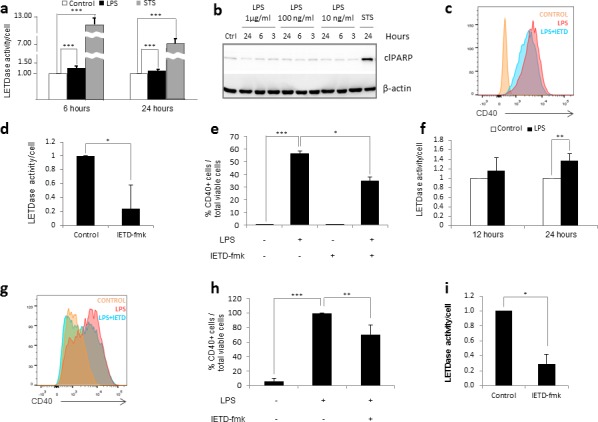
Caspase 8 regulates human monocyte activation Figure **a.-b.** THP-1 cells were treated with 1 μg/ml LPS or 0.5 μg/ml STS. a) LETDase activity was measured and normalized against the number of cells at 6 and 24 hours post-treatment. LETDase activity is displayed on a discontinuous axis. b) Analysis of cleaved PARP levels by western blot at 3, 6 and 24 post-LPS-treatment. Three LPS concentrations were tested (1 μg/ml, 100 ng/ml and 10 ng/ml). Figure **c.-e.** THP-1 cells were treated with LPS (1 μg/ml) in the presence or absence of 20 μM of IETD-fmk for 48 hours. c) Histogram representation of the mean fluorescence channel for CD40. d) LETDase activity inhibition induced by IETD-fmk. e) Quantification of CD40 positive cells. Figure **f.** Human primary monocytes were treated with 1 μg/ml LPS and LETDase activity was measured and normalized against the number of cells at 12 and 24 hours post-treatment. Figure **g.-i.** Human primary monocytes were treated with LPS (1 μg/ml) in the presence or absence of 20 μM of IETD-fmk for 48 hours. g) Histogram representation of the mean fluorescence channel for CD40. h) LETDase activity inhibition induced by IETD-fmk. i) Quantification of CD40 positive cells. Error bars represent standard deviation of the three experiments conducted. Statistical significance was assessed by Bonferroni test in e and h or Student's t test in a, d, f and i.

To replicate the environment of SIRS in vitro, we treated the human monocytic cell line THP-1 with lipopolysaccharide (LPS); a PAMP that induces monocyte activation via TLR-4 signaling. The degree of activation was assessed by the expression of CD40, given it is upregulated in inflammatory monocytes and commonly used as a biomarker for SIRS [28]. In the presence of 1μg/ml LPS, we observed a moderate increase in caspase-8 activity at 6 and 24 hours post-treatment (Figure 1a). To test whether the LPS-induced increase of caspase-8 activity was associated to apoptotic cell death, we assessed the levels of cleaved Poly (ADP-ribose) polymerase (PARP-1), a known marker of apoptotic cell death. As seen in Figure 1b, LPS treatment failed to lead to increased cleaved PARP, demonstrating that the increase in caspase-8 activity was not due to an increase in apoptotic cell death. In order to test whether the observed increase in caspase-8 activity was required for monocyte activation, we pretreated THP-1 cells with the specific caspase-8 inhibitor IETD-fmk for 30 minutes prior to LPS challenge. We found that a 70% inhibition in caspase-8 activity was able to partially reverse the LPS-induced activation (Figure 1c-1e). Based on these results, we proceeded to assess the role of caspase-8 in a more physiological model consisting of human primary monocytes purified from peripheral blood. Like THP-1 cells, primary monocytes experienced a moderate increase in caspase-8 activity in the presence of LPS (Figure 1e). Furthermore, a 70% decrease in caspase-8 activity was sufficient to partially decrease LPS-mediated activation (Figure 1f-1h). Together, these data suggest that caspase-8 is involved in the activation of monocytes, and that the inhibition of its activity can be used to modulate the intensity of this activation.

### Caspase-8 inhibition induces necroptosis of activated monocytes

**Figure 2:**
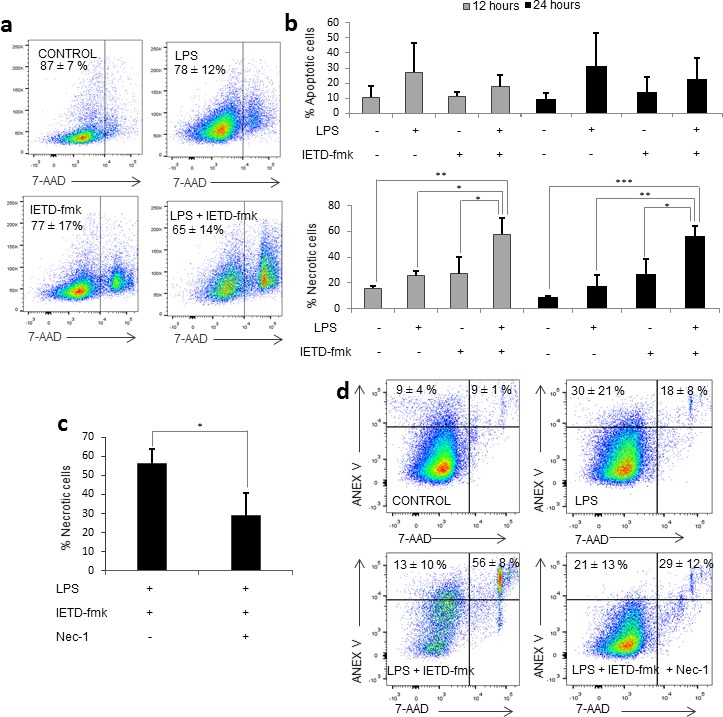
Caspase 8 inhibition induces necroptosis Primary human monocytes were treated with LPS (1 μg/ml) in the presence or absence of 20 μM of IETD-fmk and/or of 25 μM Necrostatin-1. **a.** Dot plot showing viability in each condition. **b.** Percentage of apoptotic (Annexin V+/7-AAD-) cells and necrotic (7-AAD+) cells at 12 and 24 hours post-treatment. **c.** Variation of the percentage of necrotic cells in the presence or absence of Nec-1. **d.** Dot plot showing viable, necrotic and apoptotic cells in each condition. Error bars represent standard deviation of the three experiments conducted. Statistical significance was assessed by Bonferroni test.

We noticed that the combination of LPS and IETD-fmk under the conditions stated above triggered high levels of necrotic cell death in LPS-treated primary human monocytes (Figure 2a); an effect that we had not seen in THP-1 cells (data not shown). In order to determine which cell death pathway was involved, we measured the percentage of apoptotic and necrotic cells at shorter time-points (Figure 2b). We found that there was no apoptosis prior to the induction of necrosis, meaning that the increase in necrotic cell death at 48 hours was not due to secondary necrosis. However, when we treated monocytes with the necroptosis inhibitor necrostatin-1 (Nec-1) for 15 minutes prior to LPS challenge, this was found to reverse the cell death induced by caspase-8 inhibition, suggesting that necroptosis was taking place in activated monocytes treated with caspase-8 inhibitors (Figure 2c).

### Caspase-8 inhibition decreases the levels of both pro- and anti-inflammatory cytokines associated to SIRS

**Figure 3:**
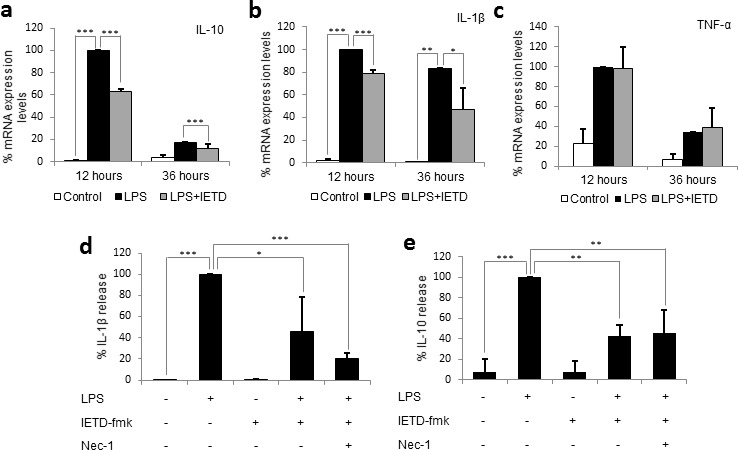
Caspase 8 inhibition decreases the production and release of SIRS-related cytokines, but not necroptosis related cytokines Primary human monocytes were treated with LPS (1 μg/ml) in the presence or absence of 20 μM of IETD-fmk and/or 25 μM of Nec-1 for 12 and 36 hours. **a.** mRNA expression levels for IL-10. **b.** mRNA expression levels for IL-1β. **c.** mRNA expression levels for TNF-α. **d.** IL-1β concentration in the supernatant. **e.** IL-10 concentration in the supernatant. Error bars represent standard deviation of the three experiments conducted. Statistical significance was assessed by Bonferroni test. IETD = IETD-fmk.

Due to the necrotic nature of necroptotic cell death, we initially hypothesized that necroptosis of primary monocytes could cause the release of DAMPs, which could activate the remaining live monocytes and thus counteract the decrease in activation achieved by caspase 8 inhibition. Therefore, we firstly measured the mRNA expression levels of the main cytokines involved in SIRS: IL-1β, IL-10 and TNF-α, which were found to increase between 5 and 100-fold at 12 hours post-LPS treatment. However, despite the induction of necroptosis, we found that the pre-treatment with IETD-fmk did not increase the mRNA expression levels of any of the three cytokines, but rather caused a significant decrease in the mRNA expression of IL-10 and IL-1β (Figure 3a-3b). The expression levels of TNF-α were not altered by IETD-fmk treatment (Figure 3c), however this may be due to the fact that TNF-α is induced during necroptosis. These findings strongly suggest that the removal by necroptosis of the excess of monocytes that proliferate during sepsis would occur without further exacerbating the innate immune response. However, the possibility remained that necroptotic monocytes might themselves have released cytokines that could initiate the SIRS cytokine cascade. Thus, we measured the concentration of SIRS-related cytokines in the media of necroptotic and non-necroptotic cell cultures and found that, in spite of the 50% necrosis induced by caspase-8 inhibition, the levels of pro-inflammatory IL-1β decreased significantly when monocytes were pre-treated with IETD-fmk prior to LPS challenge (Figure 3a). Of note, the addition of Nec-1 was found to induce a small, non-significant decrease in the concentration of IL-1β. With regards to the levels of anti-inflammatory IL-10, we found a significant reduction under caspase-8 inhibition which was maintained in the presence of Nec-1. Given that the levels of both IL-10 and IL-1β decrease under IETD-fmk treatment, we hypothesize that, unlike most conventional treatments, caspase-8 inhibition is able to counteract both phases of SIRS; pro- and anti-inflammatory. This would prevent the onset of the immunosuppressive stage and thus could be used as a potential clinical target in the treatment of SIRS.

### The effects of caspase-8 inhibition in humans can only be replicated in mice by using broad caspase inhibitors

**Figure 4:**
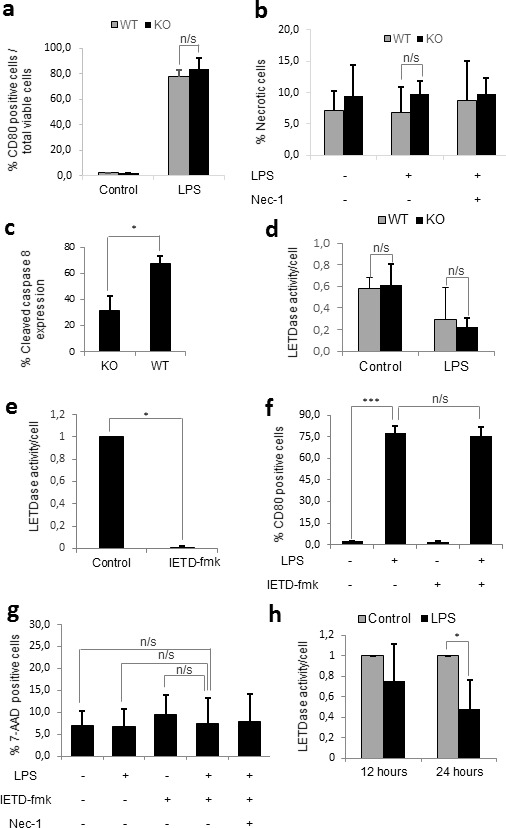
Caspase-8 alone does not regulate murine monocyte activation Figure **a.-d.** Primary monocytes from wild-type and knock-out mice were plated either alone or in the presence of 1 μg/mL LPS and/or 25 μM Necrostatin-1 for 24 hours. a) Percentage of activated (CD80 positive) monocytes. b) Percentage of necrotic (7-AAD positive) cells. c) Percentage of cleaved caspase 8 expression by flow cytometry. d) Differences in LETDase activity between wild-type and knock-out monocytes. Figure **e.-h.** Primary monocytes from wild-type mice were plated either alone or in the presence of 1 μg/mL LPS in the presence or absence of 20 μM IETD-fmk and/or 25 μM Necrostatin-1 for 24 hours e) LETDase activity inhibition induced by IETD-fmk. f) Percentage of activated (CD80 positive) monocytes g) Percentage of necrotic (7-AAD positive) cells. h) LETDase activity normalized against the number of cells. Error bars represent standard deviation of the three experiments conducted. Statistical significance was assessed by Bonferroni test in a, b, d, f and g and Student's t test in c, e and h.

In order to extrapolate our results from human monocytes to a SIRS murine model, we firstly decided to confirm whether caspase-8 also played a role in murine monocyte activation. For this purpose, bone marrow monocytes were isolated from mice lacking caspase-8 in the myeloid cell line (Caspase-8^f/f^ LysMCre^+/−^). These mice, which were generated using flox-Cre technology, have been previously documented in studies on microglial and myeloid cells [29–34]. Monocytes were purified from the bone marrow by negative selection, as the isolated monocytes have been previously shown to be equivalent to blood monocytes [35]. The monocytes from these mice were then treated with LPS for 24 hours, after which the levels of monocyte activation and necroptosis were compared to those from LPS-treated wild-type mice. Surprisingly, we were unable to find necroptosis or a decrease in monocyte activation in the cells of the knock-out mice treated with LPS (Figure 4a-4b), despite confirming a 40% decrease in cleaved caspase-8 by FACS (Figure 4c). We then measured caspase-8 activity in purified monocytes and found no difference between wild-type and knock-out mice, even in the presence of LPS. Based on these data, we conclude that the caspase-8 gene is not efficiently deleted in bone-marrow-derived monocytes from Caspase-8^f/f^ LysMCre^+/−^ mice.

Since the deletion of the caspase-8 gene in the mouse did not seem to replicate the effects of the chemical inhibition of caspase-8 achieved in humans, we decided to assess the effect of IETD-fmk on monocyte activation and necroptosis. Strikingly, despite achieving a 90% decrease in caspase-8 activity (Figure 4e), the chemical inhibition of caspase-8 neither resulted in necroptosis nor a decrease in the activation of LPS-treated murine monocytes (Figure 4f-4g). Furthermore, when we quantified the changes in caspase-8 activity induced by LPS, we found a decrease rather than the increase expected based on the results in human monocytes (Figure 4h). Given this, it appears that there may be another factor which compensates for the lack of caspase-8 activity during murine monocyte activation.

**Figure 5:**
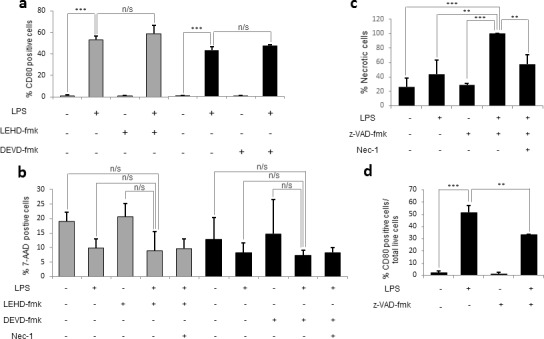
The pan-caspase inhibitor z-VAD-fmk reproduces the effects of caspase 8 inhibition in humans Murine bone-marrow monocytes were plated either alone or in the presence of 1 μg/mL of LPS and/or 20 μM of DEVD-fmk, LEHD-fmk or z-VAD-fmk and/or 25 μM Nec-1. **a.** Percentage of activated cells (CD80 positive) in the presence of DEVD-fmk and LEHD-fmk. **b.** Percentage of necrotic cells (7-AAD positive) in the presence of DEVD-fmk and LEHD-fmk. **c.** Percentage of necrotic cells in the presence of z-VAD-fmk. **d.** Percentage of activated cells in the presence of z-VAD-fmk. Error bars represent standard deviation of the three experiments conducted. Statistical significance was assessed by Bonferroni test.

It has been reported that some caspases exist only in certain species. For example, humans, but not mice, encode caspase-10 [36]. It was worth investigating whether murine monocyte activation may involve the activity of other caspases. Therefore, we assessed the effects of other caspase inhibitors on activation and necroptosis. We found that neither the specific caspase-9 inhibitor, LEHD-fmk, nor the specific caspase-3 inhibitor, DEVD-fmk, had any effect on activation or cell death when added 30 minutes prior to LPS challenge (Figure 5a-5b). However, when we pre-treated monocytes with the pan-caspase inhibitor z-VAD-fmk 30 minutes before LPS challenge, we fully reproduced the effects seen in human monocytes in terms of both activation and cell death (Figure 5c-5d). In fact, the co-treatment of monocytes with LPS and z-VAD-fmk reduced LPS-mediated activation by 20%, as well as inducing almost 60% necroptotic cell death, suggesting that other caspases may be involved in murine monocyte activation. Furthermore, given that, in both in humans and mice, a decrease in activation was never observed in the absence of necroptosis and vice versa, we hypothesize that necroptosis and a decrease in activation are linked phenotypes.

## DISCUSSION

Monocytes have been described to play an essential role in the development of SIRS, since they are capable of modulating the innate immune response [8, 37]. In line with these facts, we hypothesize that the inhibition of monocyte activation could potentially ameliorate the innate immune response and thus diminish cytokine release [15, 16], as well as depleting the excess of over-activated monocytes by necroptosis. We focused on caspase-8 as a potential regulator of monocyte activation, given its reported role in the activation and differentiation of innate immune cells [21, 38, 39]. We found that the induction of human monocyte activation with LPS induced a moderate increase in caspase-8 activity and that caspase-8 inhibition partially decreased LPS-induced activation, suggesting that caspase-8 regulates human monocyte activation. Interestingly, however, we noticed that the percentage of dead cells increased when caspase-8 was inhibited in human primary monocytes, but not THP-1 cells, activated with LPS. The death induced by the combination of LPS and IETD-fmk was exclusively necrotic, a feature which had already been described in monocytic cells under caspase inhibition [18]. Given that caspase-8 has been shown to inhibit necroptosis via RIPK1 cleavage [17, 24], we hypothesized that, under caspase-8 inhibition, RIPK1 would remain active and thus be able to induce necroptosis via association with RIPK3 and consequent MLKL activation. Under these conditions, targeting the kinase domain of RIPK1 with necrostatin-1 inhibits the interaction between RIPK1 and RIPK3 [40]. We found that necrostatin-1 prevented the death induced by the combination of LPS and IETD-fmk; confirming the occurrence of necroptosis. The absence of necroptosis in THP-1 cells could relate to recent observations that several cell lines have been shown to have alterations in the necroptosis pathway [25].

Given its pro-inflammatory nature, caused by the release of DAMPs, it was possible that necroptosis might counter the effects of caspase-8 inhibition [27]. This therefore might limit the efficacy of caspase-8 inhibition as a viable treatment for SIRS. Indeed a previous study, using necrostatin-1 treated or RIPK3^−/−^ mice has linked widespread necroptosis to the development of SIRS [41]. We hypothesized, however, that the anti-inflammatory effects of caspase-8 inhibition along with the specific depletion of activating monocytes by necroptosis would overcome the inflammatory effects of the released DAMPs. If this were the case, then the treatment of patients early with caspase-8 inhibitors would both decrease monocyte activation and lead to necroptosis of activating monocytes; cutting SIRS at the source. This would limit the resultant downstream symptoms including lymphocyte apoptosis and immunosuppression [42], which are not counteracted by other treatments. In support of this hypothesis, we detected lower levels of CD40 expression in the surviving monocytes of cultures where necroptosis had been induced with LPS and caspase-8 inhibitors. Furthermore, we found that, despite necroptosis, caspase-8 inhibition was sufficient to decrease the expression and release of IL-1β. Together, this suggests that caspase-8 inhibitors can control the inflammatory phase of SIRS. This data is in line with previous work which has shown that depletion of microglia by necroptosis can rescue the neuronal loss induced by activated microglia [43]. In addition, it has been demonstrated that L929 cells, a mouse fibrosarcoma cell line, subjected to TNF-induced necroptosis failed to induce pro-inflammatory cytokine production in macrophages [27]. A possible explanation for this is that, given active caspase-8 has been shown to be an important mediator of the release of DAMPs such as calreticulin [44], necroptosis under conditions of caspase-8 inhibition may be less inflammatory [27].

Given that active monocytes are also involved in the production of anti-inflammatory cytokines [6], we next wondered whether caspase-8 inhibition could control the anti-inflammatory phase of SIRS. We found that caspase-8 inhibition was sufficient to decrease the expression and release of the anti-inflammatory cytokine IL-10, which is involved in the immunosuppressive stage of SIRS. Thus, we hypothesize here that IETD-fmk treatment could a) counteract the initial pro-inflammatory state of SIRS, by decreasing the release of IL-1β and depleting the excess of monocytes; and b) prevent the immunosuppressive state by inhibiting the production of IL-10 in the remaining live monocytes.

Furthermore, it may also be that necroptosis itself has positive roles beyond the depletion of activated monocytes. The infusion of necrotic splenocytes prior to a sepsis challenge has been linked to reduced lymphocyte impairment and apoptosis, thus increasing survival via preventing the immunosuppressive stage of the syndrome [45]. Therefore, the presence of necrotic monocytes due to caspase-8 inhibition might also serve to stimulate the adaptive immune system and counteract the immunosuppressive stage.

It is interesting to note that caspase-8 has been involved in both priming and assembly of the NLRP3 inflammasome [46, 47]. Although our study was not designed to study the role of caspase-8 in inflammasome activation, our data may shed light into this issue. It should be highlighted that monocytes exhibit a distinctive one-step pathway of inflammasome activation that can induce robust IL-1β release in response to TLR4 stimulation alone without the need for a secondary signal [48]. Our data shows that, regardless of necroptosis, caspase-8 inhibition strongly reduced the TLR4-induced mRNA expression (priming/activation) and release of IL-1β, supporting the hypothesis that caspase-8 regulates both processes [49–51]. Further work would be required to ascertain whether caspase-8 plays an inflammasome-dependent or independent role in IL-1β processing.

**Figure 6:**
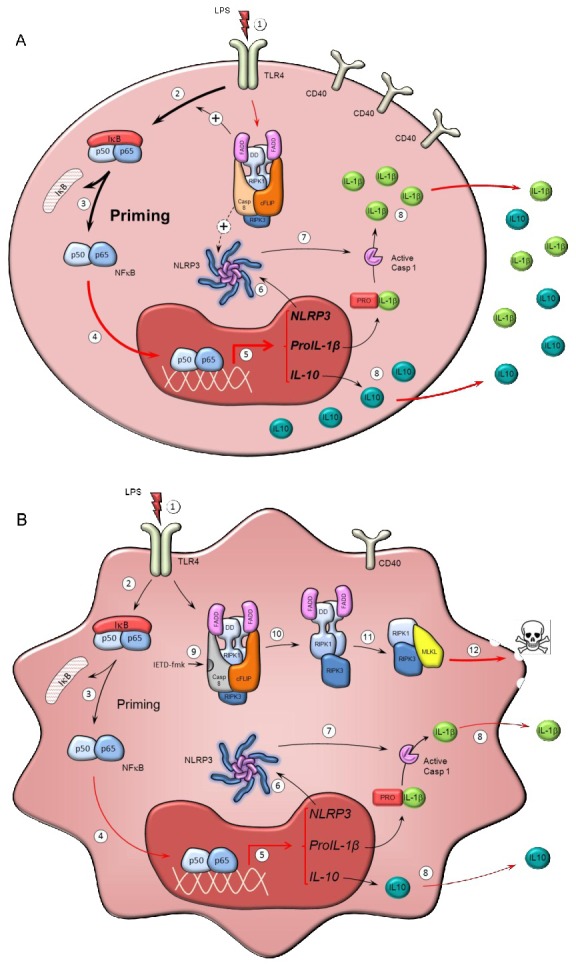
Mechanism of action of caspase-8 inhibitors in human monocytes Scheme illustrating potential mechanisms by which caspase-8 might modulate the activation of monocytes (a) and how caspase-8 inhibition may selectively kill activated monocytes under conditions of SIRS (b). **a.** TLR activation in monocytes by LPS and other PAMPs and DAMPs (1) leads to priming of the monocytes; a process mediated by NF-kB activation (2, 3) and its nuclear translocation (4), to trigger transcription of target genes (e.g. IL-1β, IL-10 and NLRP3) (5). Priming of monocytes results in the synthesis of the components of the NLRP3 inflammasome (6), whose assembly eventually leads to caspase-1 activation (7), cleavage of pro-IL1β to IL-1β and subsequent IL-1β release (8). IL1β is a major interleukin involved in innate immune responses. The ripoptosome, a molecular platform containing RIPK1, FADD, caspase-8 and cFLIP, forms in response to TLR activation in monocytes and possibly modulates the priming response, including NLRP3 activation. **b.** Effect of caspase-8 inhibition on activated monocytes. Caspase-8 activity associated to the ripoptosome prevents necroptosis by selective cleavage of RIPK1. Consequently, selective inhibition of caspase-8 by IETD-fmk (9) results in RIPK1 activation and subsequent formation of the necrosome (10 and 11), thus leading to MLKL activation (12) and the induction of necroptosis. Loss of ripoptosome assembly may result in reduced priming by the mechanism shown above, thus leading to significantly lower expression of pro-IL1β and IL-10, and reduced NLRP3 activation. Overall, caspase-8 inhibition leads to selective death of activated monocytes without exacerbating release of potential DAMPS. Red lines denote processes supported by our experimental data while black lines denote processes well-established in the literature.

Figure 6 illustrates a proposed mechanism based on our observations alongside well-established concepts in the literature. In human primary monocytes, the activation of TLR receptors leads to the expression of NF-kB-responsive genes such as IL-1β and IL-10. Our data suggests that the activation of caspase-8 alongside ripoptosome assembly takes place in response to TLR4 activation. Furthermore, it highlights the possibility that caspase-8 might have a role in the priming and activation of monocytes. The data shown here contributes to a growing body of literature about the importance of caspase-8 in the pro-inflammatory activation of microglia [21, 22], macrophages [52] and now monocytes. We propose that under caspase-8 inhibition, two events take place: i) necroptotic death of active monocytes, ii) a significant reduction in the release of interleukins involved in SIRS like IL-1β and IL-10. Furthermore, as stated above, given the essential role of the NLRP3 inflammasome in pro-IL1β processing and subsequent release, we anticipate an important role of caspase-8 in NLRP3 assembly within activated monocytes.

When trying to apply our model to murine models of SIRS, our results showed that caspase-8 may not be the only enzyme involved in murine monocyte activation and necroptosis. In contrast to humans, where the specific inhibition of caspase-8 was sufficient to decrease monocyte activation and induce necroptosis, mouse monocytes required the use of a broad caspase inhibitor to induce a similar phenotype. Interestingly, the effects of pan caspase inhibitors have already been tested in mouse models of SIRS. Hotchkiss et al. (2000) demonstrated that the intraperitoneal injection of the pan-caspase inhibitor q-VD-OPh was able to increase survival of mice under conditions of septic shock (cecal ligation and puncture) as well as decreasing the levels of pro-inflammatory cytokines [12]. Furthermore, Weber et al. (2009) showed that the use of the broad caspase inhibitor VX-166 in mice also has positive effects in two complementary models of sepsis - endotoxin shock, and cecal ligation and puncture [53]. Like Hotchkiss et al., they also observed decreased circulating levels of pro-inflammatory cytokines in VX-166 treated animals, thus supporting our observations. Although both studies attributed these effects to the prevention of lymphocyte apoptosis, it should be highlighted that lymphocyte apoptosis during SIRS is caused by monocyte over-activation [12, 53]. Thus, given our results show that treatment of murine monocytes with a pan-caspase inhibitor induces monocyte necroptosis and a decrease in monocyte activation, we hypothesize that this may be the cause of the observed decreased lymphocyte apoptosis and increased survival rate. Furthermore, given we found that the inhibition of a single protein, caspase-8, is able to reproduce the same effects in human monocytes, we hypothesize that the treatment of SIRS with caspase-8 inhibitors may provide positive results. Furthermore, the use of caspase-8 inhibitors in place of pan-caspase inhibitors may also decrease the possible side effects derived from inhibiting all caspases.

## MATERIALS AND METHODS

### General chemical material

LPS from Escherichia coli 0111:B4 (Cat. No. L5293), IETD-fmk, LEHD-fmk and necrostatin-1 were purchased from Sigma-Aldrich (St Louis, MO, USA). Z-VAD-fmk was purchased from Promega (Madison, USA). DEVD-fmk was obtained from Becton Dickinson Bioscience (San Jose, CA, USA). PBS, RPMI-1640, FBS, trypsin and antibiotics were purchased from GIBCO (InvitrogenTM, Paisely, UK). AB serum, L-glutamine, β-mercaptoethanol, PFA, methanol and bovine albumin (BSA) were purchased from Sigma-Aldrich (St Louis, MO, USA). MACS running buffer, columns and MACS separators were from Miltenyi Biotec S.L. (Bergisch Gladbach, Germany).

### Cell culture and monocyte isolation

Human monocytes were isolated from buffy-coats obtained from healthy donors in accordance with the Declaration of Helsinki. All procedures were performed based on the regulations established by the Ethical Committee of Virgen del Rocío Hospital (Seville, Spain). Monocytes were then isolated from the mononucleate cell fraction by immunomagnetic separation using CD14 microbeads (Miltenyi Biotec S.L. Bergisch Gladbach, Germany), following manufacturer's instructions, and grown in RPMI-1640 supplemented with 10% AB serum. THP-1 cells (ATCC^®^ TIB202™) were obtained from Sigma-Aldrich (88081201) and cultured in RPMI-1640 containing 10% FBS, 2 mM L-glutamine, 1 mM sodium pyruvate and 0.05 mM β-mercaptoethanol. All experimentation was carried out at Cancer Centrum Karolinska (CCK), Stockholm (Sweden), in accordance with national and European regulations.

Animal experimentation was carried out at the University of Seville, in accordance with the regulations established by in accordance with the Guidelines of the European Union Council (86/609/EU), following Spanish regulations (BOE 34/11370-421, 2013) for the use of laboratory animals and approved by the Scientific Committee of the University of Seville. Animals were kept in specific pathogen-free conditions and had free access to food and water. Caspase-8f/f C57BL/6 mice (with the CASP8allele floxed at exon 3) were generously provided by Prof. Steven M. Hedrick (University of California, San Diego) [54] (Figure 1a). C57BL/6 mice containing a Cre recombinase under the control of LysM promoter and enhancer elements, which allow its expression in the myeloid cell linage, including microglia, were obtained from Jackson Laboratories (strain name B6.129P2-Lyz2tm1(cre)Ifo/J). A cross was set up between both strains and the offspring were Casp8^f/+^.CreLysM^+/−^. These offspring were crossed with Casp8^f/f^ to generate Casp8^f/f^.CreLysM^+/−^ (myeloidCasp8KO mice) and Casp8^f/f^. CreLysM^−/−^ (WT mice). In order to make the breeding process more efficient, we crossed Casp8^f/f^. CreLysM^+/−^ with Casp8^f/f^ mice to generate a 50 % Casp8^f/f^.CreLysM^+/−^ and 50 % Casp8^f/f^.CreLysM^−/−^. Homozygous floxed Casp8^f/f^ mice were born at the predicted Mendelian ratio and were indistinguishable from littermates. Wild type, single, and double floxed targeted Casp8^−/−^ alleles were genotyped by PCR. For monocyte isolation, 6-8 week-old mice were sacrificed by cervical dislocation and the bone marrow was harvested by standard techniques. Monocytes were then purified by negative selection using the BM Monocyte Isolation Kit (Miltenyi Biotec S.L., Bergisch Gladbach, Germany) [30], following manufacturer's instructions, and cultured in RPMI-1640 supplemented with 10% FBS. To induce monocyte activation, 2×10^5^ primary human and murine monocytes were plated in flat-bottom 96-well plates. THP-1 cells were plated in flat-bottom 12-well plates at 2×10^5^ cells/well. All cells were treated with 1 μg/ml LPS and/or 20 μM of caspase inhibitor for 30 minutes before LPS, and/or 25 μM necrostatin-1 for 15 minutes before LPS.

### Caspase 8 activity assay

Changes in caspase activity in were measured using a luciferase-based assay from Promega (Madison, USA) known as Caspase-Glo (G8200 for caspase-8). Equal volumes of cells and kit component were mixed onto a 96-well plate (Costar^®^ White plates, Sigma-Aldrich, St Louis, MO, USA) and incubated for 1 h at room temperature. The plate was analyzed using a luminometer (Promega, Madison, USA) according to manufacturer's specifications and the value obtained normalized with the number of cells at harvest.

### Flow cytometry

To assess activation levels, cells were detached, washed and resuspended in PBS, and 1 × 10^5^ cells in 100 μl PBS were stained with antibodies using a 4-color combination as specified in Tables 1 and 2. Samples were incubated for 15 min in the dark and the excess of antibodies was washed with PBS at 300 g for 10 min. Prior to acquisition, 5 μl of 7-aminoactinomycin D (7-AAD) (Becton Dickinson Bioscience. San Jose, CA, USA) was added. A total of 50000 events were acquired in a FACSCantoII (Becton Dickinson Bioscience, San Jose, CA, USA) and the percentage of activated cells in the live cell population was gated using the Flow Jo software program (FlowJo LLC, OR, USA). For cell death assays, the Annexin V PE/7-AAD Kit from BD Pharmingen was used. Cells were stained with 5 μl of Annexin V and 5 μl of 7-AAD in combination to quantify the percentages of apoptotic (Annexin V+/7-AAD-) and necrotic (AnnexinV+/7-AAD+) cells. For intracellular, cleaved caspase-8 staining, bone marrow monocytes were washed with PBS and fixed with PFA and permeabilized with 90% methanol. Cells were washed and blocked (0.5% BSA in PBS), and labeled with anti-cleaved Casp8 antibody (Asp387) (Cell Signalling, Danvers, MA, USA) (D5B2) (1:800) and a secondary anti-rabbit fluorescein conjugated secondary antibody (Vector Laboratories, Burlingame, CA, USA).

**Table 1:** Antibodies used in murine monocyte activation studies

Antibody	Fluorochrome	Isotype	Volume used	Company
CD11b	FITC	Rat IgG2b	10 μl	Miltenyi Biotec S.L. Bergisch Gladbach, Germany
CD45	VioBlue	Rat IgG2bκ	10 μl	Miltenyi Biotec S.L. Bergisch Gladbach, Germany
CD80	PE	Hamster IgG2	10 μl	Miltenyi Biotec S.L. Bergisch Gladbach, Germany

**Table 2:** Antibodies for human monocyte activation studies

Antibody	Fluorochrome	Isotype	Volume used	Company
HLA-DR	APC	Mouse IgG1κ	5 μl	Becton Dickinson Bioscience San Jose CA USA
CD45	FITC	Mouse IgG1κ	10 μl	Becton Dickinson Bioscience San Jose CA USA
CD40	PE	Mouse IgG1	10 μl	Beckman Coulter Company Marselle France
CD14	APC-H7	Mouse IgG2a κ	5 μl	Becton Dickinson Bioscience San Jose CA USA

### RNA isolation, cDNA synthesis and qPCR

Total RNA was isolated with the RNeasy Mini Kit (Qiagen, Venlo, Holland) according to manufacturer's instructions. cDNA was synthesized from up to 1 μg of total RNA using the QuantiTect Reverse Transcription Kit (Qiagen, Venlo, Holland). The cDNA concentration was measured with a Nanodrop 1000 (Thermo Fischer Scientific, Nunc A/S, Kamstrupvej, Roskilde, Denmark) and 750 ng of cDNA was added to each qPCR reaction. The qPCR was performed using SensiMixPlus SYBR (Quantace, Bioline, Taunton, MA, USA) on a Mastercycler ep gradient S realplex2 (Eppendorf, Hamburg, Germany). All primer sets had similar Tm values and amplified short fragments, spanning an intron and recognizing all or most of the cDNA splicing variants described (Table 3). All primers were designed with the Universal ProbeFinder v2.44 software (Roche Applied Science; Roche Farma, S.A., Madrid, Spain) and verified by Oligo Analyzer 3.1 (Integrated DNA Technologies, Coralville, IA, USA). The amplification program was 10 minutes at 95°C followed by 45 cycles of 15 s at 95°C, 30 s at 60°C, 8 s at 72°C, and a final extension step of 5 minutes at 72°C. A melting curve from 50°C to 95°C in 30 minutes was used to verify specificity. Relative expression levels were calculated in triplicate using the comparative CT method with β-actin as a housekeeping gene.

**Table 3:** Primers for RT-qPCR

*PRIMER*	*SEQUENCE (5′- 3′)*	*Tm (°C)*
IL-10 F	AAGACCCAGACATCAAGGCG	66.4
IL-10 R	CAGGGAAGAAATCGATGACAGC	66.9
IL-1β F	AAAGCTTGGTGATGTCTGGTC	63.1
IL-1β R	GGACATGGAGAACACCACTTG	64.4
TNFα F	TGTTGTAGCAAACCCTCAAGC	64.0
TNFα R	TATCTCTCAGCTCCACGCCA	66.1
βACT F	GGCCAGGTCATCACCATTG	66.2
βACT R	CACAGGACTCCATGCCCAG	66.4

### Enzyme-linked immunosorbent assay

Cytokine concentration was quantified in cell culture supernatants using IL-1β (human) ELISA kit (Enzo Life Sciences Farmingdale, NY, USA) for the determination of IL-1β, and IL-10 (human) ELISA kit (Enzo Life Sciences, Farmingdale, NY, USA) for IL-10. All the experiments were performed following manufacturer's procedures. Absorbance was read at 450 nm, in a Synergy HT plate reader (BioTek Winooski, VT, USA).

### Statistical analysis

All data are presented as mean ± standard deviation/range of either absolute values or percentages. Statistical analyses were performed using IBM SPSS Statistics 19 software. Statistical significance was assessed by Student's *t*-test or one-way ANOVA followed by the Bonferroni test. In all the tests, P values were *P* < 0.05 (*), *P* < 0.01 (**) and *P* < 0.001 (***).

## References

[1] Ward PA, Bosmann M (2012). A Historical Perspective on Sepsis. American Journal of Pathology.

[2] Uppu DSSM, Ghosh C, Haldar J (2015). Surviving sepsis in the era of antibiotic resistance: Are there any alternative approaches to antibiotic therapy?. Microbial Pathogenesis.

[3] Esteban A, Frutos-Vivar F, Ferguson ND, Penuelas O, Lorente JA, Gordo F, Honrubia T, Algora A, Bustos A, Garcia G, Rodriguez Diaz-Reganon I, Ruiz de Luna R (2007). Sepsis incidence and outcome: Contrasting the intensive care unit with the hospital ward. Critical Care Medicine.

[4] Kang J, Kim S, Cho H, SM L (2015). DAMPs activating innate immune responses in sepsis. Ageing Res Rev.

[5] Feldman N, Rotter-Maskowitz A, Okun E (2015). DAMPs as mediators of sterile inflammation in aging-related pathologies. Ageing Res Rev.

[6] Stearns-Kurosawa DJ, Osuchowski MF, Valentine C, Kurosawa S, Remick DG (2011). The Pathogenesis of Sepsis. Annual review of pathology.

[7] Fingerle G, Pforte A, Passlick B, Blumenstein M, Strobel M, Zieglerheitbrock HWL (1993). THE NOVEL SUBSET OF CD14+/CD16+ BLOOD MONOCYTES IS EXPANDED IN SEPSIS PATIENTS. Blood.

[8] Serbina NV, Jia T, Hohl TM, Pamer EG (2008). Monocyte-Mediated Defense Against Microbial Pathogens. Annu Rev Immunol.

[9] Lewis DH, Chan DL, Pinheiro D, Armitage-Chan E, Garden OA (2012). The Immunopathology of Sepsis: Pathogen Recognition, Systemic Inflammation, the Compensatory Anti-Inflammatory Response, and Regulatory T Cells. Journal of Veterinary Internal Medicine.

[10] Bosmann M, Ward PA (2013). The inflammatory response in sepsis. Trends in Immunology.

[11] Weber GF, Chousterman BG, He S, Fenn AM, Nairz M, Anzai A, Brenner T, Uhle F, Iwamoto Y, Robbins CS, Noiret L, Maier SL, Zoennchen T, Rahbari NN, Schoelch S, Ameln AK-v (2015). Interleukin-3 amplifies acute inflammation and is a potential therapeutic target in sepsis. Science.

[12] Hotchkiss RS, Chang KC, Swanson PE, Tinsley KW, Hui JJ, Klender P, Xanthoudakis S, Roy S, Black C, Grimm E, Aspiotis R, Han Y, Nicholson DW, Karl IE (2000). Caspase inhibitors improve survival in sepsis: a critical role of the lymphocyte. Nature Immunology.

[13] Sharawy N, Lehmann C (2015). New directions for sepsis and septic shock research. Journal of Surgical Research.

[14] Dellinger RP, Levy MM, Rhodes A, Annane D, Gerlach H, Opal SM, Sevransky JE, Sprung CL, Douglas IS, Jaeschke R, Osborn TM, Nunnally ME, Townsend SR, Reinhart K, Kleinpell RM, Angus DC (2013). Surviving Sepsis Campaign: International Guidelines for Management of Severe Sepsis and Septic Shock, 2012. Intensive Care Medicine.

[15] Matsuda A, Jacob A, Wu R, Aziz M, Yang W-L, Matsutani T, Suzuki H, Furukawa K, Uchida E, Wang P (2012). Novel Therapeutic Targets for Sepsis: Regulation of Exaggerated Inflammatory Responses. Journal of Nippon Medical School.

[16] Antonopoulou A, Giamarellos-Bourboulis EJ (2010). Immunomodulation in sepsis: state of the art and future perspective. Immunotherapy.

[17] Oberst A, Green DR (2011). It cuts both ways: reconciling the dual roles of caspase 8 in cell death and survival. Nature Reviews Molecular Cell Biology.

[18] Sordet O, Rebe C, Plenchette S, Zermati Y, Hermine O, Vainchenker W, Garrido C, Solary E, Dubrez-Daloz L (2002). Specific involvement of caspases in the differentiation of monocytes into macrophages. Blood.

[19] Jacquel A, Benikhlef N, Paggetti J, Lalaoui N, Guery L, Dufour EK, Ciudad M, Racoeur C, Micheau O, Delva L, Droin N, Solary E (2009). Colony-stimulating factor-1-induced oscillations in phosphatidylinositol-3 kinase/AKT are required for caspase activation in monocytes undergoing differentiation into macrophages. Blood.

[20] Guery L, Benikhlef N, Gautier T, Paul C, Jego G, Dufour E, Jacquel A, Cally R, Manoury B, Vanden Berghe T, Vandenabeele P, Droin N, Solary E (2011). Fine-tuning nucleophosmin in macrophage differentiation and activation. Blood.

[21] Burguillos MA, Deierborg T, Kavanagh E, Persson A, Hajji N, Garcia-Quintanilla A, Cano J, Brundin P, Englund E, Venero JL, Joseph B (2011). Caspase signalling controls microglia activation and neurotoxicity. Nature.

[22] Kavanagh E, Rodhe J, Burguillos MA, Venero JL, Joseph B (2014). Regulation of caspase-3 processing by cIAP2 controls the switch between pro-inflammatory activation and cell death in microglia. Cell Death & Disease.

[23] Silke J, Rickard J, Gerlic M (2015). The diverse role of RIP kinases in necroptosis and inflammation. Nature Immunology.

[24] Vandenabeele P, Galluzzi L, Vanden Berghe T, Kroemer G (2010). Molecular mechanisms of necroptosis: an ordered cellular explosion. Nature Reviews Molecular Cell Biology.

[25] de Almagro MC, Vucic D (2015). Necroptosis: Pathway diversity and characteristics. Seminars in Cell & Developmental Biology.

[26] Murphy JM, Czabotar PE, Hildebrand JM, Lucet IS, Zhang J-G, Alvarez-Diaz S, Lewis R, Lalaoui N, Metcalf D, Webb AI, Young SN, Varghese LN, Tannahill GM, Hatchell EC, Majewski IJ, Okamoto T (2013). The Pseudokinase MLKL Mediates Necroptosis via a Molecular Switch Mechanism. Immunity.

[27] Kaczmarek A, Vandenabeele P, Krysko DV (2013). Necroptosis: The Release of Damage-Associated Molecular Patterns and Its Physiological Relevance. Immunity.

[28] Yang J, Zhang L, Yu C, Yang X-F, Wang H (2014). Monocyte and macrophage differentiation: circulation inflammatory monocyte as biomarker for inflammatory diseases. Biomarker research.

[29] Ye M, Iwasaki H, Laiosa CV, Stadtfeld M, Xie H, Heck S, Clausen B, Akashi K, Graf T (2003). Hematopoietic stem cells expressing the myeloid lysozyme gene retain long-term, multilineage repopulation potential. Immunity.

[30] Kavanagh E, Angel Burguillos M, Carrillo-Jimenez A, Jose Oliva-Martin M, Santiago M, Rodhe J, Joseph B, Luis Venero J (2015). Deletion of caspase-8 in mouse myeloid cells blocks microglia pro-inflammatory activation and confers protection in MPTP neurodegeneration model. Aging (Albany NY).

[31] Wieghofer P, Knobeloch KP, Prinz M (2015). Genetic targeting of microglia. Glia.

[32] Goldmann T, Wieghofer P, Muller PF, Wolf Y, Varol D, Yona S, Brendecke SM, Kierdorf K, Staszewski O, Datta M, Luedde T, Heikenwalder M, Jung S, Prinz M (2013). A new type of microglia gene targeting shows TAK1 to be pivotal in CNS autoimmune inflammation. Nat Neurosci.

[33] Madisen L, Zwingman TA, Sunkin SM, Oh SW, Zariwala HA, Gu H, Ng LL, Palmiter RD, Hawrylycz MJ, Jones AR, Lein ES, Zeng H (2010). A robust and high-throughput Cre reporting and characterization system for the whole mouse brain. Nat Neurosci.

[34] Immig K, Gericke M, Menzel F, Merz F, Krueger M, Schiefenhovel F, Losche A, Jager K, Hanisch UK, Biber K, Bechmann I (2015). CD11c-positive cells from brain, spleen, lung, and liver exhibit site-specific immune phenotypes and plastically adapt to new environments. Glia.

[35] Oliva-Martin MJ, Ignacio Sanchez-Abarca L, Carrillo-Jimenez A, Perez-Simon JA, Venero JL (2015). Evaluation of a method for murine monocyte isolation by bone marrow depletion. Analytical Biochemistry.

[36] Sakamaki K, Satou Y (2009). Caspases: evolutionary aspects of their functions in vertebrates. Journal of Fish Biology.

[37] Karlmark KR, Tacke F, Dunay IR (2012). Monocytes in health and disease - Minireview. European journal of microbiology & immunology.

[38] Kang TB, Ben-Moshe T, Varfolomeev EE, Pewzner-Jung Y, Yogev N, Jurewicz A, Waisman A, Brenner O, Haffner R, Gustafsson E, Ramakrishnan P, Lapidot T, Wallach D (2004). Caspase-8 serves both apoptotic and nonapoptotic roles. Journal of Immunology.

[39] Rebe C, Cathelin S, Launay S, Filomenko R, Prevotat L, L'Ollivier C, Gyan E, Micheau O, Grant S, Dubart-Kupperschmitt A, Fontenay M, Solary E (2007). Caspase-8 prevents sustained activation of NF-kappa B in monocytes undergoing macrophagic differentiation. Blood.

[40] Fuchs Y, Steller H (2015). Live to die another way: modes of programmed cell death and the signals emanating from dying cells. Nat Rev Mol Cell Biol.

[41] Duprez L, Takahashi N, Van Hauwermeiren F, Vandendriessche B, Goossens V, Vanden Berghe T, Declercq W, Libert C, Cauwels A, Vandenabeele P (2011). RIP Kinase-Dependent Necrosis Drives Lethal Systemic Inflammatory Response Syndrome. Immunity.

[42] de Pablo R, Monserrat J, Prieto A, Alvarez-Mon M (2014). Role of Circulating Lymphocytes in Patients with Sepsis. Biomed Research International.

[43] Fricker M, Vilalta A, Tolkovsky AM, Brown GC (2013). Caspase Inhibitors Protect Neurons by Enabling Selective Necroptosis of Inflamed Microglia. Journal of Biological Chemistry.

[44] Panaretakis T, Kepp O, Brockmeier U, Tesniere A, Bjorklund A-C, Chapman DC, Durchschlag M, Joza N, Pierron G, van Endert P, Yuan J, Zitvogel L, Madeo F, Williams DB, Kroemer G (2009). Mechanisms of pre-apoptotic calreticulin exposure in immunogenic cell death. Embo Journal.

[45] Hotchkiss RS, Chang KC, Grayson MH, Tinsley KW, Dunne BS, Davis CG, Osborne DF, Karl IE (2003). Adoptive transfer of apoptotic splenocytes worsens survival, whereas adoptive transfer of necrotic splenocytes improves survival in sepsis. Proc Natl Acad Sci U S A.

[46] Antonopoulos C, Russo H, El Sanadi C, Martin B, Li X, Kaiser W, Mocarski E, Dubyak G (2015). Caspase-8 as an Effector and Regulator of NLRP3 Inflammasome Signaling. J Biol Chem.

[47] Kang T-B, Yang S-H, Toth B, Kovalenko A, Wallach D (2013). Caspase-8 Blocks Kinase RIPK3-Mediated Activation of the NLRP3 Inflammasome. Immunity.

[48] Carta S, Tassi S, Pettinati I, Delfino L, Dinarello CA, Rubartelli A (2011). The Rate of Interleukin-1 Secretion in Different Myeloid Cells Varies with the Extent of Redox Response to Toll-like Receptor Triggering. J Biol Chem.

[49] Bossaller L, Chiang PI, Schmidt-Lauber C, Ganesan S, Kaiser WJ, Rathinam VAK, Mocarski ES, Subramanian D, Green DR, Silverman N, Fitzgerald KA, Marshak-Rothstein A, Latz E (2012). FAS mediates non-canonical IL-1 and IL-18 maturation via caspase-8 in a Rip3-independent manner(). Journal of immunology (Baltimore, Md : 1950).

[50] Maelfait J, Vercammen E, Janssens S, Schotte P, Haegman M, Magez S, Beyaert R (2008). Stimulation of Toll-like receptor 3 and 4 induces interleukin-1 maturation by caspase-8. J Exp Med.

[51] Shenderov K, Riteau N, Yip R, Mayer-Barber KD, Oland S, Hieny S, Fitzgerald P, Oberst A, Dillon CP, Green DR, Cerundolo V, Sher A (2014). ER stress licenses macrophages to produce mature IL-1 in response to TLR4 stimulation through a caspase-8- and TRIF-dependent pathway. Journal of immunology (Baltimore, Md : 1950).

[52] Gurung P, Anand PK, Malireddi RK, Vande Walle L, Van Opdenbosch N, Dillon CP, Weinlich R, Green DR, Lamkanfi M, Kanneganti TD (2014). FADD and caspase-8 mediate priming and activation of the canonical and noncanonical Nlrp3 inflammasomes. Journal of immunology (Baltimore, Md : 1950).

[53] Weber P, Wang P, Maddens S, Wang PSH, Wu R, Miksa M, Dong W, Mortimore M, Golec JMC, Charlton P (2009). VX-166: a novel potent small molecule caspase inhibitor as a potential therapy for sepsis. Critical Care.

[54] Ch'en IL, Tsau JS, Molkentin JD, Komatsu M, Hedrick SM (2011). Mechanisms of necroptosis in T cells. Journal of Experimental Medicine.

